# Cared and uncared populations: understanding unmet care needs of older adults (65+) across different social care systems in Europe

**DOI:** 10.1007/s10433-023-00760-3

**Published:** 2023-04-29

**Authors:** Mariana Calderón-Jaramillo, Pilar Zueras

**Affiliations:** 1 Universitat Autònoma de Barcelona, Barcelona, Catalunya Spain; 2 Centre d’Estudis Demogràfics (CED-CERCA) – Cerdanyola del Valles, Barcelona, Catalunya Spain; 3 Institute for Social and Economic Research, University of Essex, Colchester, UK

**Keywords:** Social care systems, Ageing, Unmet care needs, Care provision, Europe

## Abstract

Population care needs are dynamic. They change throughout individuals’ life courses and are related to the population structure. These needs are particularly demanding during population ageing and may vary depending on how societies cope with them. In this study, we explored the unmet social care needs of individuals in twelve European countries with different social care systems. We used data from the seventh wave of the Survey of Health, Ageing and Retirement in Europe (SHARE) to conduct a cross-sectional study of individuals aged 65 and over with care needs (*n* = 7136). Unmet care needs were measured from an absolute approach. We fitted binomial regression models to explain the relative importance of individuals’ characteristics, health status and different social care systems on unmet needs. The absolute measure shows that 53.02% of the analytical sample faced unmet care needs as they reported limitations and did not receive help. The prevalence of unmet care needs is higher for men than women and for younger than older individuals. Furthermore, we found that individuals living in Mediterranean social care systems have the highest prevalence of these unmet needs. This analysis contributes to the ongoing debate about the challenges posed by ageing populations and their relationship with care.

## Background

Care is a basic need of human beings throughout their life. As anthropological and philosophical work has suggested, care constitutes individuals’ personhood (Buch 2015) and acts in the foundations of society itself (Fisher and Tronto 1990). The most paradoxical aspect of care, however, is that it is usually recognized because of the lack of it. People usually feel they are not receiving enough care when they stop being cared for or when new care needs emerge. Furthermore, demographic dynamics fundamentally impact social care demand and supply (Spijker et al. 2022). For instance, care needs are not the same between populations in the earlier stages of the demographic transition, characterized by high fertility rates where children take up most of the care and the social services, in comparison with those in the fourth stage of the transition that are facing ageing processes and challenges regarding caring for older people (Bom and Stöckel 2021; Rechel et al. 2013).

European countries are forerunners in this ageing process that will affect many countries worldwide (Vaupel and Kistowski 2008). Therefore, Europe is a critical scenario for understanding population ageing effects on care provision, policies, and welfare systems. Previous literature has highlighted that care is affected by its gendered provision, as is mainly given by women (Schmid et al. 2012; Uccheddu et al. 2019; Young and Grundy 2008); the central role played by the family and informal care provision (Pickard et al. 2007; Tennstedt et al. 1993); and new changes in the design and use of social services (Cantor 1991; Davey 2017; Spijker and Zueras 2020).

Some authors have suggested that we are facing a care crisis driven by demographic dynamics leading to population ageing and changes in family trajectories, household units, and social and economic transformations (Pérez Orozco 2021). Discussions about care provision have also underlined how it is affected by policy changes (Pfau-Effinger 2005) and social perceptions about ageing and support that usually shape specific care systems to help people with disabilities and facing limitations in daily life. However, in many societies, some individuals are not receiving the support they need and are facing unmet care needs that can negatively affect their health, well-being, and life expectancy.

In this article, we aim to analyse the unmet care needs experienced by people aged 65 and over within twelve European countries. We examined the socio-demographic characteristics of middle-age and older adults with care needs and estimated the prevalence of unmet care needs in the following social care systems: the Mediterranean, characterized by family-based care provision; the Nordic, where care provision is strongly linked to welfare-state services; the Western, where care provision is articulated between informal and formal care provision, also including the participation of private providers; and, the Eastern, which used to be based on ‘familialist’ care provision but has undergone various transformations since the fall of the Berlin Wall.

### Different typologies for understanding social care systems

Social care is conceptualized as the coexistence of informal and formal care activities that addresses three primary needs: socialization, activities of daily living and personal needs related to severe disability (Cantor 1991). The differences between social care systems are related mainly to the way informal and formal care is organized. For example, family-centred systems rely primarily on informal care, whereas welfare-state centred systems emphasize the availability of formal care through its provision by people who are not relatives.

Theoretically, the configuration of social care systems relates to values (Pfau-Effinger 2005), ancient family systems (Reher 1998), religion (Damiani et al. 2011), and the structural socioeconomic context (Ariaans et al. 2021) that have shaped care provision itself as well as public policies related to it. The starting point for exploring unmet care needs is the recognition that social care systems may fail to provide universal coverage, access, and funding for individuals’ care needs. This idea also emphasizes that the relationship between formal and informal care provision is not always virtuous, that the availability of one of these types of care does not guarantee the availability of the other, and that access to both does not necessarily lead to all care needs being met; for example, there may be times of the day when the individuals has no one to help them, or certain tasks for which they do not get the help they need.

The literature on social care systems mainly focuses on childcare and infants’ care needs; meanwhile, the one referred to the care for the older population is based on different typologies. These have been built according to theoretical or empirical perspectives. The theoretical approach focuses on the configuration of the welfare state within Europe, where care systems fall on the spectrum of family-centred care (Hrast et al. 2020) and social care-based services provided by the welfare state (Bergmark et al. 2000; Pfau-Effinger 2005). This theoretical framework refers especially to service provision and articulation between informal and formal care provision.

On the other hand, the empirical approach has constructed different typologies of social care systems using statistical methods such as clustering and principal components analyses. Previous evidence has focused on OECD, high-income and middle-income countries and has emphasized diverse aspects of care provision like service availability, public expenditure, care demand, performance, and, regulation (Ariaans et al. 2021; Damiani et al. 2011). Despite the importance of this approach, one of its main limitations is that the demographic dynamics in care provision remain barely explored. In this article, we focused on the theoretical typology to explore unmet care needs in countries where social care systems have been shaped by a long-term policy history.

### Measuring unmet care needs

Underlying the measurement of unmet care needs is the discussion about social care services and how informal and formal care are articulated through policies, public institutions, households, and families (Broese van Groenou & de Boer 2016; Uccheddu et al. 2019). However, research on this topic has stressed the challenges of measuring unmet care needs among the ageing population (Allen et al. 2014; Bień et al. 2013; Dunatchik et al. 2019; Stein et al. 2020). These difficulties by and large occur because surveys do not usually include enough information about care provision and the quality of care received. Consequently, its analysis should be done through indirect estimations based on questions about experiencing functional limitations that affect the performance of daily life activities.

Evidence on the subject has identified different dimensions of these functional limitations and distinguishes between mobility, Activities of Daily Living (ADLs) and Instrumental Activities of Daily Living (IADLs) (Ćwirlej-Sozańska et al. 2019; Mlinac & Feng 2016; Wolinsky et al. 2011). The definition used here is based on previous work by Vlachantoni’s (2011), where unmet care needs from an absolute approach refer to the type and amount of support received by someone who reports functional limitations (mobility, ADL and IADL) and is, consequently, assumed to be in need of help but does not report receiving it. Previous evidence has shown the importance of demographic and socioeconomic circumstances on people’s needs and unmet care needs and has emphasized that socioeconomic variables like housing tenure and education level may explain the experience of unmet needs (Maplethorpe et al. 2015; Vlachantoni 2019). It has also called attention to the relationship between unmet needs, health conditions (McGilton et al. 2018), and types of limitations faced (Mlinac & Feng 2016).

In this article, we explore two hypotheses about the unmet care needs of people over 65 based on previous literature. Firstly, due to women’s greater longevity and likelihood of being widowed and living alone (Delbès et al. 2006), as well as the fact that those with worst health and financial circumstances have less access to care provision outside home (Dupraz et al. 2020), we hypothesized that women in the older age group, in poor health and with low educational attainment (Momtaz et al. 2012), would be those that are most likely to face unmet needs (Hypothesis A). Second, in terms of issues related to the functioning of social care systems, there are concerns about the availability of informal care provision and its limits in meeting the increasing demand for care (Pickard et al. 2007; Tennstedt et al. 1993), hence, we hypothesized that the propensity to have unmet care needs would be higher among middle-aged and older adults living in countries with family-centred social care systems (Mediterranean) than in countries with other types of state participation (Hypothesis B).

## Data and methods

### Data

This cross-sectional study uses data from the seventh wave of the Survey of Health, Ageing and Retirement in Europe (SHARE), collected in 2017 (Börsch-Supan 2022). The SHARE provides harmonized longitudinal data through eighth waves about individuals aged 50 and older, and their partners, from 28 participant countries (27 European countries plus Israel). The eighth wave of SHARE, with more recent data is currently available, but it was collected during the pandemic of COVID-19 when many changes in older adults’ lives and care provision at the household level took place (Lebrasseur et al. 2021). In the seventh wave, the module about physical health included questions about functional limitations and care received by individuals (Börsch-Supan et al. 2013). However, the relevant questions for this study were not available in all countries.

**Fig. 1:**
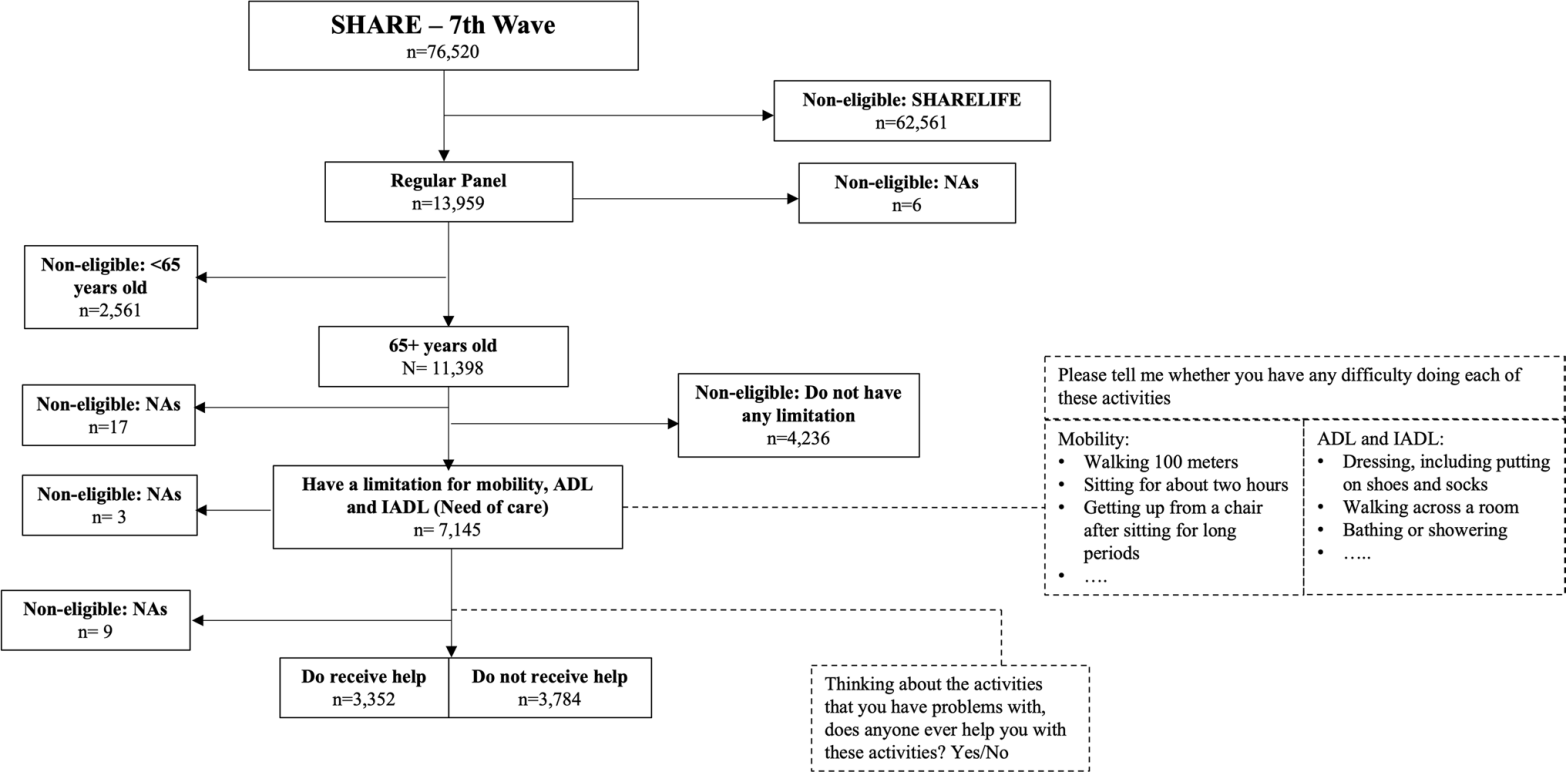
Flow chart for the selection of the analytical sample. *Note* ADL: Activities of Daily Living; IADL: Instrumental Activities of Daily Living. *Source* Survey of Health Ageing and Retirement in Europe, seventh wave (2017)

We selected 12 countries based on the availability of the studied variables concerning facing limitations in daily life and receiving help or not for dealing with these limitations. The analytical sample was composed of individuals who reported having limitations in performing at least one activity related to mobility, ADL or IADL. Figure 1 includes the flowchart and questions to illustrate the selection process of the analytical sample. This sample was composed of 7,136 individuals with complete information for the questions about coping any of the previously mentioned limitations and care received.

### Analytical strategy

The analysis of unmet care needs consisted of two steps. Firstly, we measured the percentage of people with absolute unmet care needs by type of limitation and analysed these measures by age, gender, and social care system. Secondly, we conducted a multivariate analysis regressing the binary dependent variable of absolute unmet need for social care (0 = received care; 1 = did not receive care) considering socio-demographic and health variables: gender, age group, educational level, marital status, housing tenure, living arrangements, self-rated health, chronic disease, and type of limitations. These variables are ex ante harmonized through the SHARE, which are also harmonized with similar surveys from other countries like the ELSA (England) and the HRS (US) (Börsch-Supan 2017), the variables are measured indirectly through individuals’ responses and were selected based on available evidence on unmet care needs, in particular on Vlachantoni’s previous study of England with data from the ELSA (2019). Finally, given the purpose of this study, the variable accounting for the European countries’ clusters by social care systems was also included.

Regression models were built using a forward method and were aligned with the two hypotheses. Model one included the individuals’ demographic and socioeconomic variables, and the second model added the macro variable identifying the social care system. Model three again considered individuals’ socio-demographic characteristics and included information on the type of limitation, to better understand its relationship with unmet care needs. Model four added all the health variables, and model five adjusted for having children as an indicator of potential availability of care outside the household. Finally, model six included all the previous variables and, again, the social care system of the country of residence.

### Variables

As mentioned above, the dependent variable was the absolute unmet need for social care, measured through the question related to help received by individuals reporting any mobility, ADL and IADL limitation. Participants were asked about these limitations through two questions referring to 25 activities, 10 for mobility and 15 combining ADL (6 limitations) and IADL (9 limitations). For mobility limitations, the question was: “Please look at card 36. Please tell me whether you have any difficulty doing each of the everyday activities on this card. Exclude any difficulties that you expect to last less than three months.”^1^ On the other hand, for measuring ADL and IADL, the survey asked: “Please tell me if you have any difficulty with these activities because of a physical, mental, emotional or memory problem. Again, exclude any difficulties you expect to last less than three months.”^2^ In addition, for those who report having problems with any of these types of activities, the survey includes the following question: “Thinking about the activities that you have problems with, does anyone ever help you with these activities?”.

Independent variables were included as follows. Age was aggregated into three categories (65–74, 75–84 and 85+); the education level was harmonized through ISCED 1997 classification and grouped into low (until primary school), mid (secondary education), and high education (college and above); even though ISCED 2011 is also included in the SHARE, this variable presented higher proportions of missing values than the ISCED 1997. Housing tenure was also regrouped into three categories: (i) owner, (ii) tenant and (iii) other; this last category includes members of a cooperative, subtenant and rent-free.

Self-rated health was treated as binary, distinguishing between good health (excellent, very good or good) and poor health (fair or poor self-rated health). Besides, given the information available, we followed the approach used by Spijker and Zueras (2020) and combined the type of functional limitations to create a variable that captures the degree of severity depending on the type of limitations reported: (i) facing only mobility limitations (for those who reported any mobility limitation but no limitations in performing IADLs and ADLs), (ii) those who reported limitations in one ADL and/or any IADL, (iii) those who reported limitations in two or more ADLs. Even though the Global Activity Limitation Indicator (GALI) has been validated as a severity measure and is also included in the SHARE, it does not provide detailed information about the type of limitation faced by individuals, moreover, the way in which it is included in the questionnaire does not allow to directly connect it with the measure of unmet care needs that we used.

Finally, countries were grouped into four theoretical regions according to their social care system following a welfare-state configurations typology (Pfau-Effinger 2005). The Mediterranean social care system includes Spain, Greece, and Italy; the Nordic considers Sweden and Denmark; the Western has Germany, France, Austria, Switzerland, and Belgium; and the Eastern is composed of the Czech Republic and Poland. Table 1 displays the composition of the analytical sample by age, sex, and type of limitations by the social care system.

**Table 1:** Descriptive statistics of the analytical sample by social care system

	Mediterranean		Eastern		Western		Nordic		Total sample	
	*n* = 2662	%	*n* = 1160	%	*n* = 2364	%	*n* = 950	%	*n* = 7136	%
Age group										
65–69	543	20.4	289	24.9	511	21.6	170	17.9	1513	21.2
70–74	588	22.1	301	25.9	497	21.0	226	23.8	1612	22.6
75–79	569	21.4	225	19.4	465	19.7	195	20.5	1454	20.4
80–84	510	19.2	183	15.8	406	17.2	160	16.8	1259	17.6
85+	452	17.0	162	14.0	485	20.5	199	20.9	1298	18.2
Gender										
Female	1616	60.7	726	62.6	1460	61.8	615	64.7	4417	61.9
Type of limitation										
Only mobility	1456	54.7	627	54.1	1223	51.7	508	53.5	3814	53.4
One ADL and/or any IADL	852	32.0	356	30.7	873	36.9	327	34.4	2408	33.7
Two or more ADL	354	13.3	177	15.3	268	11.3	115	12.1	914	12.8

ADL: Activities of Daily Living; IADL: Instrumental Activities of Daily Living

Source: Survey of Health Ageing and Retirement in Europe, seventh wave (2017)

### Sensitivity analysis

We conducted an alternative analysis exploring different aggregations of marital status and living arrangements to understand how unmet care needs were related to the availability of potential informal care within households. Marital status was grouped in two different ways. First, we considered three categories: (i) married or with a registered partner, (ii) ever married, and (iii) never married; secondly, four categories distinguishing: (i) married or with a registered partner, (ii) divorced or separated, (iii) never married, and (iv) widowed. However, none of these variables showed significance and were removed to avoid multicollinearity with the living arrangements variable.

We extended sensitivity analysis by grouping living arrangements in two different ways. In the first place, the following three arrangements: Living arrangements distinguished people (i) living with their partner, either with or without other people, (ii) living alone, and (iii) living with other people but the partner. Secondly, as (i) living alone, (ii) living as a couple, with the partner only, (iii) living with one or more relatives and non-relatives. The results including this second way of coding living arrangements, by which we take into account the availability of care provided by the partner, showed a lower level of statistical significance than the first one included in the final models.

The severity variable aimed to explore how the number and type of limitations explained the experience of unmet care needs. Before including it, we fit the models with the specific limitations (ADL, IADL, and mobility) and also fitted three different models for individuals by each specific limitation but the results were very similar to those presented here and did not include the number of limitations, which is related to the amount of help needed, so we used the severity variable with the categories described earlier, which considered both the type and number of limitations. Finally, we also analysed results by including countries instead of social care systems, which showed the internal coherence of the Mediterranean social care system and the differences within the other groups, especially for the Nordic and Eastern countries.

## Results

We present two types of results: First, the descriptive analysis of the analytical sample focusing on the prevalence of unmet care needs from an absolute approach and the demographic characteristics of those with any of these needs; second, binomial regression models, which illustrate how individuals’ demographic and economic characteristics and health status explain the experience of unmet care needs as well as its relationship with specific social care systems in Europe.

### Who needs care?

**Fig. 2:**
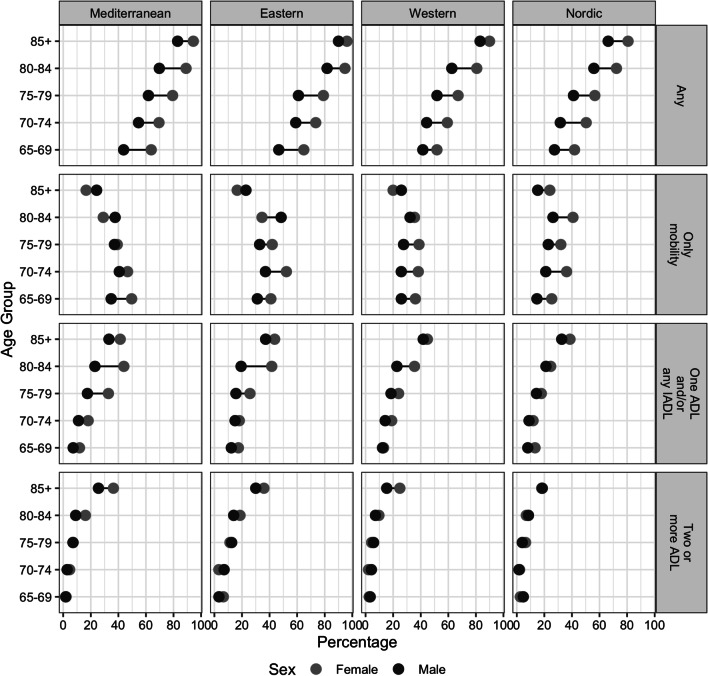
Percentage of people 65 + with functional limitations by type of limitation, age, sex, and social care system. *Note* ADL: Activities of Daily Living; IADL: Instrumental Activities of Daily Living; All: presents any type of limitation on these activities. *Source* Survey of Health Ageing and Retirement in Europe, seventh wave (2017)

People with any limitation (mobility, ADLs and IADLs) were considered to be at risk of having unmet care needs. Figure 2 presents the prevalence of each type of limitation among women and men by age group and social care system. In the four social care systems analysed, women have more limitations of any type (69.67%; CI 68.54–70.80%) than men (53.74%; CI 52.37–55.11%); these percentages are also higher in the Eastern region (70.43%; CI 68.22–72.63%) for individuals with any type of limitations and for specific type of limitations.

As expected, the prevalence of care needs is higher and more severe in older age groups. The most common type of limitation below age 85 is to experience only mobility difficulties, while limitations for performing one ADL and/or any IADL are the most common for those aged 85 and over. Having only mobility limitations shows the highest prevalence across the sample (33.46%, CI 32.60–34.32%), exceeding 15% in all the age-sex groups. Also, smaller percentages of this population face the other two types of limitations, and differences between men and women regarding the prevalence of limitations related to ADLs and IADLs are minor in the younger age groups and in the oldest age group. However, the gender gap is larger in the Mediterranean social care system, and in the Nordic social care systems for people aged 85+ .

**Fig. 3:**
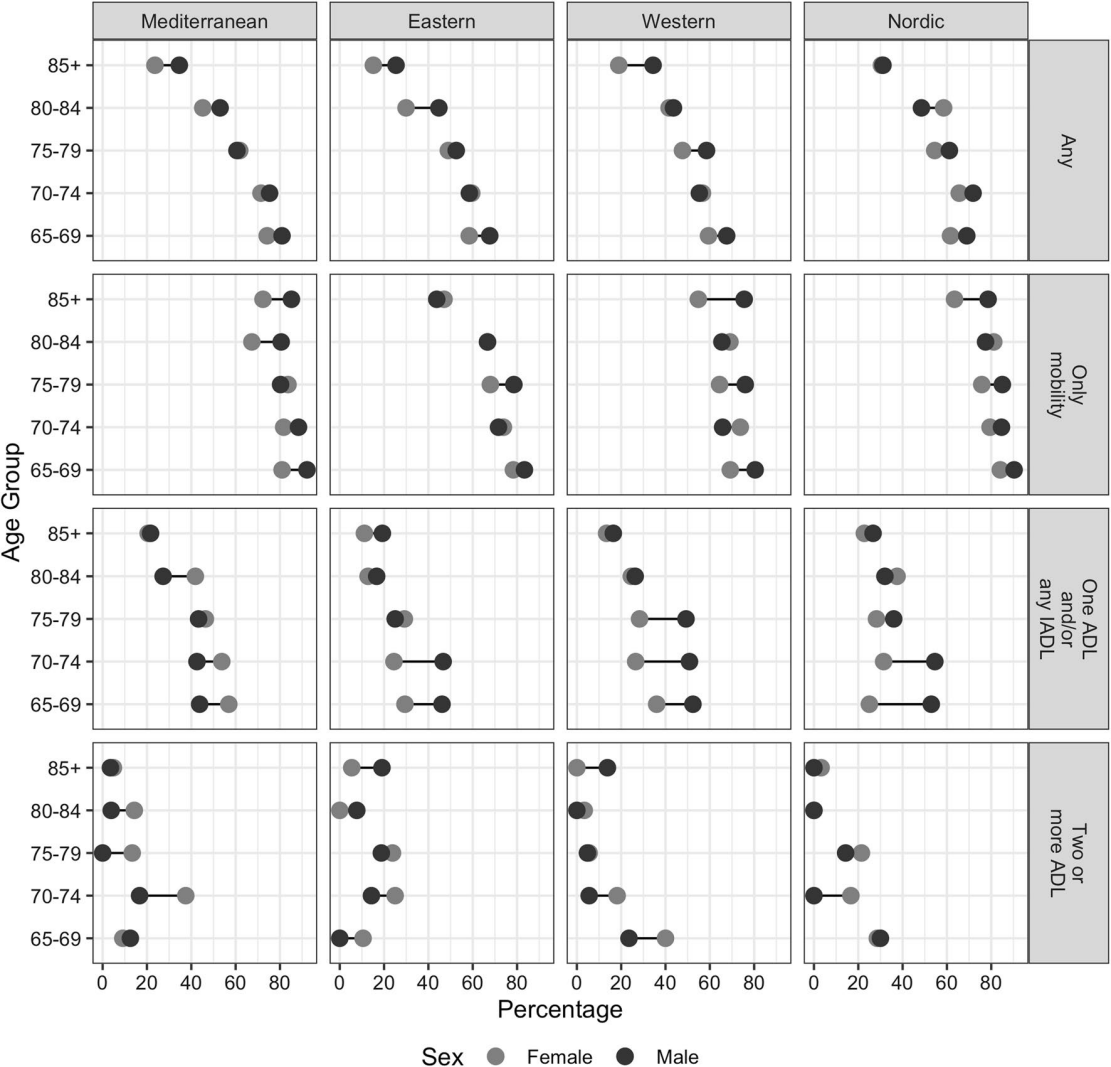
Percentage of people 65+ facing unmet care needs from an absolute approach by type of limitation, age, sex, and social care system. *Note* ADL: Activities of Daily Living; IADL: Instrumental Activities of Daily Living; All: presents any type of limitation on these activities. *Source* Survey of Health Ageing and Retirement in Europe, seventh wave (2017)

Relating to the prevalence of unmet care needs, from the absolute approach, 53.03% (CI 51.87–54.18%) of the individuals in the analytical sample (*n* = 7136) dealt with these. Therefore, more than half of the population who reported at least one limitation did not receive any help. Figure 3 shows the results by social care system, age, sex, and type of limitation. The main trend is that the percentage of people with any limitation experiencing unmet care needs is lower in the older age groups, and, with some exceptions for the age-sex groups. In general, proportions are higher for men (56.75%; CI 54.90–58.61%) than women (50.73%; CI 49.26–52.21%), even though the latter experience more limitations than the other.

In general, individuals with any limitation from the Mediterranean group have higher percentages of unmet care needs (56.87%; CI 57.00–60.73%), which is also the trend by age and gender when compared with the other three groups. Moreover, Fig. 3 shows the relevance of mobility limitations in the experience of this circumstance because the higher percentages of unmet care needs are experienced by people with only mobility limitations. Additionally, percentages of individuals with unmet care needs that face two or more ADL are lower when compared with the other types of limitations (less than 40% for all the analysed combinations of age and sex groups).

Percentages of unmet care needs also varied across countries, Table 2 presents percentages of individuals with unmet care needs for those with any limitations and by type of limitation. Overall, the trend is that more than 30% of the population with any limitation is experiencing unmet care needs. Greece has the highest percentage (68.60%; CI 63.10–68.60%), and the Czech Republic the lowest (31.82%; CI 27.85–35.80%). However, less than 25% of people with two or more ADL limitations make front of unmet care needs in all countries. For example, in Czech Republic and Austria, less than the 3% of people that has two or more ADL limitations have unmet needs (respectively 1.51%; CI − 1.43–4.46 and 2.26; CI − 2.33–7.33%), while in France, they are the 10.34% (CI 2.51–18.18%). At the same time, these percentages are higher for people facing one ADLs and/or one or more IADLs difficulties, ranging between 14.81% (CI 9.75–19.90%) in Czech Republic and 42.64% (CI 36.60–48.67%) in Spain.

**Table 2:** Percentage of people 65 + with an unmet care need from an absolute approach by country and type of limitation

Social Care System	Country	Any		Only mobility		One ADL and/or any IADL		Two or more ADL	
		*n*	%	*n*	%	*n*	%	*n*	%
Mediterranean	Spain	340	49.49 [45.75–53.23]	220	80.00 [75.27–84.72]	110	42.64 [36.60–48.67]	10	6.49 [2.60–10.39]
	Greece	750	65.85 [63.09–68.60]	599	82.50 [79.74–85.27]	144	41.86 [36.65–47.07]	7	10.14 [3.02–17.27]
	Italy	477	57.06 [53.70–60.41]	379	83.30 [79.87–86.72]	82	32.80 [26.98–38.62]	16	12.21 [6.60–17.82]
Western	Germany	179	43.77 [38.96–48.57]	150	60.00 [53.93–66.07]	27	25.23 [17.00–33.46]	2	3.84 [− 1.38–9.07]
	France	316	57.04 [52.91–61.16]	227	81.36 [76.79–85.93]	83	38.25 [31.78–44.71]	6	10.34 [2.50–18.18]
	Belgium	347	42.16 [38.79–45.54]	251	67.29 [62.53–72.05]	89	25.36 [20.80–29.91]	7	7.07 [2.01–12.12]
	Austria	153	50.33 [44.70–55.95]	125	81.17 [74.99–87.34]	27	24.55 [16.50–32.58]	1	2.50 [− 2.34–7.34]
	Switzerland	135	49.27 [43.35–51.19]	102	61.08 [53.68–68.47]	29	32.95 [23.13–42.78]	4	21.05 [2.72–39.38]
Nordic	Sweden	309	62.05 [57.79–66.31]	243	82.09 [77.73–86.46]	60	40.00 [32.16–47.84]	6	11.54 [2.85–20.22]
	Denmark	212	46.90 [43.30–51.50]	163	76.89 [71.21–82.56]	44	24.66 [18.49–31.22]	5	7.93 [1.26−14.61]
Eastern	Poland	398	62.97 [59.21–66.73]	316	89.27 [86.04–92.49]	62	37.13 [29.80–44.45]	20	18.01 [10.87–25.17]
	Czech Republic	168	31.81 [27.85–35.79]	139	50.92 [44.99–56.85]	28	14.81 [9.75–19.88]	1	1.51 [− 1.43–4.46]

ADL: Activities of Daily Living; IADL: Instrumental Activities of Daily Living. Confidence intervals, in squared brackets, were estimated based on the z value for 95% confidence (1.96) and standard errors from the analytical sample

*Source* Survey of Health Ageing and Retirement in Europe, seventh wave (2017)

### The experience of unmet care needs: individuals vs social care systems

Table 3 summarizes the results of six regression models. Similar results were observed between the first two models, which refer mainly to demographic and economic characteristics (model 1) and social care systems (model 2). In models 3 to 6, we observed the importance of health status in explaining unmet care needs, in these models the variables of self-reported health and chronic disease where included and both showed statistical significance (*p* < 0.001) for these coefficients in the three versions of the models. According to the statistics used (Akaike and Bayesian indexes of goodness of fit, AIC and BIC), model 6 had the best fit. It included demographic, economic and health variables, having children (a potential source of care), and social care systems.

**Table 3:** Binomial logistic regressions for estimating unmet care needs in different social care systems

	Model 1	Model 2	Model 3	Model 4	Model 5	Model 6
Intercept	0.418***	0.612***	1.298*	1.966***	2.434***	3.650***
	[0.344, 0.507]	[0.493, 0.758]	[1.038, 1.623]	[1.552, 2.492]	[1.782, 3.329]	[2.611, 5.112]
Age						
85+ (Ref.)	1	1	1	1	1	1
65–74	5.555***	5.461***	3.030***	3.127***	3.113***	3.009***
	[4.768, 6.485]	[4.681, 6.385]	[2.545, 3.611]	[2.620, 3.737]	[2.608, 3.720]	[2.517, 3.602]
75–84	2.984***	2.929***	2.029***	2.105***	2.100***	2.032***
	[2.575, 3.465]	[2.525, 3.403]	[1.714, 2.404]	[1.775, 2.500]	[1.771, 2.494]	[1.712, 2.415]
Gender						
Female (Ref.)	1	1	1	1	1	1
Male	1.239***	1.227***	1.366***	1.427***	1.413***	1.410***
	[1.115, 1.378]	[1.103, 1.365]	[1.212, 1.541]	[1.264, 1.612]	[1.251, 1.597]	[1.248, 1.595]
Living arrangements						
Partner in household (Ref.)	1	1	1	1	1	1
Living alone	1.048	1.062	1.322***	1.321***	1.277***	1.302***
	[0.933, 1.178]	[0.945, 1.195]	[1.157, 1.511]	[1.155, 1.513]	[1.112, 1.468]	[1.132, 1.497]
In other arrangements	0.607***	0.596***	0.784*	0.831	0.816+	0.801+
	[0.496, 0.741]	[0.486, 0.729]	[0.622, 0.987]	[0.658, 1.047]	[0.647, 1.030]	[0.633, 1.012]
Housing tenure						
Owner (Ref.)	1	1	1	1	1	1
Tenant	1.002	1.112	1.034	1.082	1.081	1.205*
	[0.863, 1.164]	[0.952, 1.298]	[0.874, 1.225]	[0.912, 1.284]	[0.911, 1.283]	[1.009, 1.440]
Other	0.890	0.985	0.894	0.945	0.953	1.030
	[0.756, 1.047]	[0.834, 1.164]	[0.744, 1.075]	[0.785, 1.138]	[0.792, 1.148]	[0.851, 1.247]
Level of Education						
High (Ref.)	1	1	1	1	1	1
Mid	0.713***	0.716***	0.756***	0.769**	0.772**	0.767**
	[0.622, 0.818]	[0.622, 0.823]	[0.648, 0.882]	[0.657, 0.899]	[0.660, 0.902]	[0.654, 0.900]
Low	0.806**	0.673***	1.064	1.116	1.124	0.931
	[0.700, 0.929]	[0.578, 0.782]	[0.906, 1.250]	[0.948, 1.315]	[0.954, 1.325]	[0.782, 1.108]
Social care system						
Mediterranean (Ref.)		1				1
Nordic		0.745***				0.784*
		[0.629, 0.883]				[0.643, 0.956]
Western		0.559***				0.527***
		[0.491, 0.636]				[0.453, 0.613]
Eastern		0.588***				0.632***
		[0.504, 0.685]				[0.528, 0.756]
Self-reported health						
Good health (Ref.)				1	1	1
Poor health				0.708***	0.707***	0.662***
				[0.626, 0.800]	[0.625, 0.800]	[0.584, 0.750]
Chronic disease						
No (Ref.)				1	1	1
Yes				0.569***	0.570***	0.598***
				[0.500, 0.647]	[0.501, 0.649]	[0.524, 0.682]
Type of limitation						
Only mobility (no ADL nor IADL)			1	1	1	1
One ADL and/or any IADL			0.165***	0.188***	0.187***	0.189***
			[0.146, 0.186]	[0.166, 0.212]	[0.166, 0.211]	[0.167, 0.214]
Two or more ADL			0.039***	0.052***	0.051***	0.051***
			[0.030, 0.049]	[0.040, 0.066]	[0.040, 0.065]	[0.039, 0.065]
Children						
No (Ref.)					1	1
Yes					0.804*	0.807*
					[0.653, 0.990]	[0.653, 0.995]
Num.Obs	7136	7136	7136	7136	7136	7136
AIC	9187.2	9101.8	7583.7	7436.8	7434.6	7364.0
BIC	9256.0	9191.2	7666.1	7533.1	7537.7	7487.7
F	− 4.583.617	− 4.537.907	− 3.779.830	− 3.704.423	− 3.702.311	− 3.663.999
RMSE	68.272	56.848	148.446	129.183	119.964	99.875

+*p* < 0.1, *p < 0.05, **p < 0.01, ***p < 0.001

Odds ratio are reported with its confidence intervals in squared brackets. ADL: Activities of Daily Living; IADL: Instrumental Activities of Daily Living

*Source* Survey of Health Ageing and Retirement in Europe, seventh wave (2017)

In all models, younger people (65–74) presented higher risks of dealing with unmet care needs than the 85+ group, but this difference showed a statistically significant reduction of the odds ratio after adjusting for health status from 8.908 in model 1 to 3.007 in model 6, this reduction is smaller but also noticeable in the age group from 75 to 84 from 4.971 in model 1 to 2.032 (see Table 4). Also, men were statistically significant (*p* < 0.001) at higher risk of experiencing that situation than women in all models. The odds of facing unmet care needs differed depending on living arrangements: living with other than the partner reduced the risk of experiencing it (results were statistically significant with different p values for all the models but the fourth one).

**Table 4:** Comparison between model 1 (rescaled to the variance) and model 6

	Estimate	OR	Std	Error	*z* value	Pr( >|z|)
*Age*						
65–74						
Model 1	2.187	8.908	0.093	23.404	< 2.2e − 16	***
Model 6	1.101	3.007	0.091	12.049	< 2.2e − 16	***
Difference	1.085		0.062	17.385	< 2.2e − 16	***
75–84						
Model 1	1.404	4.071	0.088	157.977	< 2.2e − 16	***
Model 6	0.709	2.032	0.087	80.729	6,87E − 13	***
Difference	0.695		0.057	120.377	< 2.2e − 16	***
*Gender*						
Male						
Model 1	0.294	1.342	0.062	47.326	2,22E − 03	***
Model 6	0.343	1.409	0.062	54.882	4,06E − 05	***
Difference	− 0.049		0.034	− 14.43	0.149	
*Living arrangements*						
Living alone						
Model 1	0.108	1.114	0.068	15.784	0.114	
Model 6	0.263	1.301	0.071	37.012	0	***
Difference	− 0.155		0.042	− 36.778	0	***
In other arrangements						
Model 1	− 0.644	0.525	0.118	− 54.378	5,39E − 05	***
Model 6	− 0.221	0.802	0.119	− 18.539	0.063	
Difference	− 0.422		0.074	− 57.034	1,18E − 05	***
*Housing Tenure*						
Tenant						
Model 1	0.009	1.009	0.087	0.105	0.915	
Model 6	0.186	1.204	0.09	20.602	0.039	
Difference	− 0.177		0.053	− 33.129	0	*
Other						
Model 1	− 0.143	0.867	0.095	− 15.067	0.131897	
Model 6	0.029	1.029	0.097	0.3034	0.761593	
Difference	− 0.172		0.058	− 29.578	0.003099	**
*Level of education*						
Mid						
Model 1	− 0.431	0.650	0.08	− 53.516	8,72E − 05	***
Model 6	− 0.265	0.767	0.081	− 32.531	0.001	**
Difference	− 0.166		0.042	− 39.333	8,38E − 02	***
Low						
Model 1	− 0.243	0.784	0.083	− 29.196	0.003	**
Model 6	− 0.071	0.931	0.088	− 0.803	0.421	
Difference	− 0.171		0.052	− 32.761	0.001	**

*Source* Survey of Health Ageing and Retirement in Europe, seventh wave (2017)

The educational attainment showed similar results across models, suggesting that being low- and middle-educated was associated with lower risks of experiencing unmet care needs than higher-educated individuals. However, differences for individuals in the lower levels of education became non-significant after controlling for health variables (models 3 to 6). Regarding health variables, first, the type of limitation showed that those with ADLs and IADLs were less at risk of experiencing unmet care needs than those with mobility limitations alone (*p* < 0.001). In this line, individuals with self-reported chronic diseases and poor health were not that exposed to experience unmet care needs than those without chronic disease and good health (*p* < 0.001). In addition, having children is associated with a more consistent satisfaction of individuals’ care needs when compared to those who do not have children (*p* < 0.001).

Finally, the models showed that the risk of suffering unmet care needs is lower for individuals in other social care systems than the Mediterranean. This risk was lower in the final model for the Western region (0.527, *p* < 0.001) and higher in the Nordic one (0.784, *p* < 0.05); however, smaller *p* values were observed in the Nordic group (*p* < 0.001 vs. *p* < 0.05). Refined analysis including countries instead of regions, revealed considerable heterogeneity within the analysed social care systems, particularly in the Eastern and Nordic social care systems. In the former, Czech Republic had lower than expected odds ratios, and in the later, Sweden odds ratios were not statistically significant different from Spain. In contrast, countries in the Mediterranean and Western regions had more homogeneous results (Table 5).

**Table 5:** Odd ratios of model 6 using countries instead of grouping by social care systems

Social care system	Country	OR
Mediterranean	Spain (Ref.)	1
	Greece	0.729 [0.570–0.933]
	Italy	0.832 [0.644–1.073]
Western	Germany	0.262 [0.190–0.361]
	France	0.684 [0.514–0.909]
	Belgium	0.298 [0.227–0.389]
	Austria	0.494 [0.347–0.703]
	Switzerland	0.287 [0.200–0.411]
Nordic	Sweden	0.763 [0.560–1.039]
	Denmark	0.404 [0.296–0.552]
Eastern	Poland	1.353 [1.020–1.796]
	Czech Republic	0.149 [0.110–0.203]

Confidence intervals are provided in squared brackets

*Source* Survey of Health Ageing and Retirement in Europe, seventh wave (2017)

## Discussion

This study aimed to understand the unmet care needs of people aged 65+ from different social care systems in twelve European countries. Results showed that the most vulnerable individuals (with poor health, chronic disease, older age group, and women) are at lower risk of experiencing unmet care needs, rejecting Hypothesis A. This is consistent with previous evidence from England, which suggested that men were at a higher risk of experiencing these (Vlachantoni 2019) and that older people with poor health were more likely to report receiving care (Maplethorpe et al. 2015). These results are probably due to social awareness of the care and social support needed by older people with health problems, indicating the importance of social imaginaries.

In contrast, we observed that people living in countries with Mediterranean social care systems are at a higher risk of having unmet care needs than in other systems, in line with Hypothesis B. These results are indicative of the diverse approaches within social care systems, as well as of social awareness about the urgent care needs required by older adults. Additionally, results show that family-centred systems may face more challenges in meeting individuals’ needs due to its dependence on the availability of family members willing and able to provide care (Tennstedt et al. 1993), and these may be changing as women’s engagement in the labour market increases. For example, a study in Spain showed that the willingness to care for the older family members was lower among women with a high level of education and doing paid work (Zueras et al. 2018). Previous studies have also emphasized that ageing due to demographic changes poses challenges on the availability of informal because of low fertility rates and increases in the percentage of dependent elders who are childless (Spijker and Zueras 2020).

The main contribution of this study is its comparative approach to the analysis of this largely unexplored issue. Our findings show that unmet care needs change depending on the social care systems of the countries where older people live. Despite previous research has shown differences in social care systems between regions and countries (Ariaans et al. 2021; Dunatchik et al. 2019; Pfau-Effinger 2005), to our knowledge this is the first study comparing unmet care needs between different social care systems. Results spotlighted that the demographic characteristics like the age group and gender, were associated with unmet care needs, i.e., a higher risk was found for men than women and for younger than older age groups. In line with previous research, findings showed that the type of limitation explained the risk of facing unmet care needs, in our results individuals with only mobility limitations faced lower risk, meanwhile others have shown that the chance of suffer them is more strongly associated to ADL (Vlachantoni 2019). Living with other people in the household is associated with a lower risk, which may indicate that care is being provided by someone other than the couple, although previous research has shown that partners are still the main informal care providers (Kaschowitz and Brandt 2017; Uccheddu et al. 2019; Young and Grundy 2008).

In addition, people who live with someone other than a partner (compared with living alone or with a partner with or without another person) and who are neither owners nor tenants of the house in which they live are less likely to have unmet care needs. Previous evidence on the subject comes from England, where it was estimated that about 55% of older individuals with ADL, 24% of people with an IADL difficulty, and 80% of people with a mobility limitation have unmet care needs based on ELSA (Vlachantoni 2019). In contrast, this study found lower percentages of unmet care needs by each type of limitation, even for the population with only mobility difficulties, for whom the highest percentage was found in Greece. Nevertheless, these results are not fully comparable as the estimation comes from similar but not equivalent questions and filters in the analysed surveys (Ashokkumar et al. 2012).

However, further research about the relationship between these unmet needs and different social care systems is still needed; through the sensitivity check of the models, it was visible that there are differences within the groups of Nordic and Eastern countries. For instance, the results for Sweden and Poland may be explained by recent changes in care policies in these two countries. In the Swedish case, changes during the last three decades have been orientated towards enhancing voluntary choices and individuals’ involvement in their own care; however, these measures are taking place in a context where the second demographic transition may affect the availability of care provision by children and partners (Edlund and Lövgren 2022; Moberg 2021). Meanwhile, Poland’s history is characterized by an essential differentiation between hospice-palliative care, which emerged in the seventh decade of the last century, and home care (Krakowiak 2020), gaps between these two ways of care provision may reflect the lower quality of informal care provided in Poland when compared to the other countries (Dobrzyn-Matusiak et al. 2014).

In any case, this study has some limitations related to the sample and the measure that we used. The most relevant limitation is posed by the assumption behind measuring unmet care needs, which supposes that individuals facing any limitation, in fact, need help, even though some of them may be able to cope with these limitations without the support of a caregiver. Another limitation comes from the small sample size and lack of representativeness of the analysis by countries, which is why we used groups of countries based on theoretical typologies of social care systems, despite there are internal differences between the countries that are part of the Nordic and the Eastern social care systems. How to construct typologies of social care systems is still an ongoing debate. Previous evidence says that there may be more appropriate criteria than a regional approach (Ariaans et al. 2021; Damiani et al. 2011). Nevertheless, this study based its theoretical typology on previous work about welfare state configuration (Pfau-Effinger 2005).

Some relevant aspects come from using SHARE data to measure unmet care needs. While studies based on the ELSA usually ask if someone facing a limitation is receiving the help needed for performing a specific activity (e.g., bathing or eating), the SHARE asks this after all the questions about limitations for performing these activities are asked, which makes it impossible to know the specific activities for which individuals are facing these unmet needs. Likewise, we cannot truly know if the individual needs help to cope with the limitations that s/he is facing. This problem can only be solved by adding a new question in the survey that directly ask if the person needs care from other to perform these activities.^3^ Still, the main value of this study lies in its comparative nature, which makes it possible to provide an empirical estimate of unmet care needs in 12 countries and to gain insights into the differences between social care systems at the regional level, which may be useful for policy makers interested in care demand and provision in ageing societies.

## Conclusions

Care provision within ageing scenarios make front of challenges in assuring people’s rights and well-being. This article suggests that individuals from older age groups and those in poorer health and worse functioning conditions face more negligible risks of experiencing unmet care needs. This scenario could indicate that social care systems meet the most pressuring needs: they are reactive but not preventive because they do not consider the future effects of unmet care needs on individuals’ morbidity, well-being, and physical and mental health. Also, living arrangements respond to higher needs of care and are effective in supplying at least some of it; in spite of that, whether this is sufficient, or the most appropriate care, should also be a matter of investigation. Care is a basic need that changes through life courses and poses challenges to ageing populations, particularly in those societies based on family-centred care provision. More information and research are needed to examine current and future responses to the actual care demands to leave no one behind.

## Footnotes

1 Activities on card 36 included: i) Walking 100 m; ii) Sitting for about two hours; iii) Getting up from a chair after sitting for long periods; iv) Climbing several flights of stairs without resting; v) Climbing one flight of stairs without resting; vi) Stopping, kneeling, or crouching; vii) Reaching or extending your arms above shoulder level; viii) Pulling or pushing large objects like a living room chair; ix) Lifting or carrying weights over 10 pounds/5 kilos, like a heavy bag of groceries; and x) Picking up a small coin from a table.

2 The activities asked for were: i) Dressing, including putting on shoes and socks; ii) Walking across a room; iii) Bathing or showering; iv) Eating, such as cutting up your food; v) Getting in or out of bed; vi) Using the toilet, including getting up or down; vii) Using a map to figure out how to get around in a strange place; viii) Preparing a hot meal; ix) Shopping for groceries; x) Making telephone calls; xi) Taking medications; xii) Doing work around the house or garden; xiii) Managing money, such as paying bills and keeping track of expenses; xiv) Leaving the house independently and accessing transportation services; and xv) Doing personal laundry. Activities from i to vi refer to ADL, while from vii to xv are related to IADL.

3 Following the SHARE wording it can be formulated as: “Thinking about the activities that you have problems with, do you need help or support from someone else for performing these activities? Yes/No”.
